# Patient-reported outcomes in Hodgkin lymphoma trials: a systematic review

**DOI:** 10.3389/fonc.2024.1353101

**Published:** 2024-03-13

**Authors:** Esther Natalie Oliva, Tatyana Ionova, Edward Laane, Mario Csenar, Julia Schroer, Karolin Behringer, Ina Monsef, Annika Oeser, Nicole Skoetz, Sam Salek

**Affiliations:** ^1^ Hematology Unit, Grande Ospedale Metropolitano Bianchi Melacrino Morelli, Reggio di Calabria, Italy; ^2^ Quality of Life Monitoring Unit, Saint Petersburg State University Hospital, Saint Petersburg, Russia; ^3^ Hematology-Oncology Clinic, Tartu University, Tartu, Estonia; ^4^ Evidence-Based Medicine, Department I of Internal Medicine, Faculty of Medicine and University Hospital Cologne, University of Cologne, Cologne, Germany; ^5^ Center for Integrated Oncology Aachen Bonn Cologne Duesseldorf, Department I of Internal Medicine, Faculty of Medicine and University Hospital Cologne, University of Cologne, Cologne, Germany; ^6^ School of Life and Medical Sciences, University of Hertfordshire, Hatfield, United Kingdom

**Keywords:** Hodgkin lymphoma, patient-reported outcomes, Quality of Life, PRO instrument, psychometric testing, systematic review, validation studies

## Abstract

**Background:**

Lymphoma treatment can lead to long-term consequences such as fatigue, infertility and organ damage. In clinical trials, survival outcomes, clinical response and toxicity are extensively reported while the assessment of treatment on quality of life (QoL) and symptoms is often lacking.

**Objective:**

We evaluated the use and frequency of patient-reported outcome (PRO) instruments used in randomized controlled trials (RCTs) for Hodgkin lymphoma (HL) and their consistency of reporting.

**Methods:**

MEDLINE, CENTRAL and trial registries for RCTs investigating HL were systematically searched from 01/01/2016 to 31/05/2022. Following trial selection, trial, patient characteristics and outcome data on the use of PRO measures (PROMs) and reporting of PROs using a pre-defined extraction form were extracted. To assess reporting consistency, trial registries, protocols and publications were compared.

**Results:**

We identified 4,222 records. Following screening, a total of 317 reports were eligible for full-text evaluation. One hundred sixty-six reports of 51 ongoing/completed trials were included, of which 41% of trials were completed and 49% were ongoing based on registry entries. Full-text or abstract were available for 33 trials. Seventy percent of trials were conducted in the newly diagnosed disease setting, the majority with advanced HL. In 32 trials with published follow-up data, the median follow-up was 5.2 years. Eighteen (35%) completed/ongoing trials had mentioned PRO assessment in registry entries, protocol or publications. Twelve trials (67%) had published results and only 6 trials (50%) reported on PROs in part with the exception of 1 trial where PROs were evaluated as secondary/exploratory outcome. The most referenced global PROM was the EORTC-QLQ-C30 (12 studies), the EQ-5D (3 studies) and the FACT-Neurotoxicity (3 studies). FACT-Lymphoma, a disease-specific PROM for non-HL was mentioned in one ongoing trial. None of the trials referenced the EORTC QLQ-HL27, another disease-specific PROM developed specifically for HL patient’s QoL assessment.

**Discussions:**

Only one-third of RCTs in HL report PROs as an outcome and only half present the outcome in subsequent publications, showcasing the underreporting of PROs in trials. Disease-specific PROMs are underutilized in the assessment of QoL in HL patients. Guidance on the assessment of PROs is needed to inform on comprehensive outcomes important to patients.

**Systematic review registration:**

https://www.crd.york.ac.uk/PROSPERO/display_record.php?RecordID=391552, identifier CRD42023391552.

## 1 Introduction

Hodgkin lymphoma (HL) is a lymphoid neoplasm that involves the lymph nodes and lymphatic system (1–3). It represents approximately 30% of all lymphomas. HL accounts for 0.5% of all new cases of cancer worldwide with an average age-standardized rate of 0.98 per 100,000 individuals annually (4). While the absolute incidence of HL has remained unchanged, it is one of the most frequent cancers diagnosed in adolescent age and young adults with 2–3 cases per 100,000 annually in developed countries. A bimodal incidence for HL by age is observed, with a first peak seen in adolescence and young adulthood (aged 15–40) and a second peak after the age of 55 (5). Still, young adults are most often affected. Male predominance of HL is also observed (male:female ratio of 1.5:1), however this is not seen in the nodular sclerosis subtype of HL (6). According to The WHO classification, HL is divided into two main types: classic HL and nodular lymphocyte-predominant HL (7) with the majority (˜95%) diagnosed with classic HL (8). A hallmark of classic HL is the presence of Reed-Sternberg cells (in an inflammatory background), whereas in nodular lymphocyte-predominant HL Reed-Sternberg cells are absent but it is characterized by the presence of lymphocyte-predominant cells (i.e. popcorn cells). Patients with HL frequently present with painless localized or wide-spread lymphadenopathy, B symptoms that includes profound weight loss, unexplained high fevers and drenching night sweats (9). B symptoms are common in about one-third of patients and are generally occur more frequently in HL stage 3 to 4, mixed cellularity and lymphocyte depleted HL subtypes (10, 11). They contribute to the worsening of patients’ well-being. In addition, alcohol induced pain in lymph nodes and chronic pruritus are known to be common disease symptoms (11). Fatigue is another frequently reported symptom associated with HL (12).

Due to stage-adapted treatment, including chemotherapy with or without consolidation radiotherapy, HL has become one of the best curable malignancies in adults in the past few decades (3). It is now curable in at least 80% of patients younger than 60 years of age (13, 14). In every patient with newly diagnosed HL there is an extremely high likelihood of being cured with the appropriate treatment. In patients with more advanced disease (stages IIB–IV), the main challenge is to increase the proportion of patients with durable remissions while reducing the possibility of long-term side effects. Despite the high cure rate being obtained with initial therapy, about 5% to 10% of patients with HL are refractory to initial treatment, and 10% to 30% of patients will relapse even after achieving an initial complete remission (15, 16). The treatment of relapsed or refractory disease requires additional exposure to toxicity through salvage regimens, radiotherapy, and potentially high-dose therapy with autologous hematopoietic cell transplant (15, 16). It is increasingly recognized that CD30- and PD-1-targeted therapies play an important role in the treatment of HL. The increase in the number of long-term survivors of HL has led to the increasing importance of late sequelae and quality of life (QoL) in these patients. Noteworthy, novel treatment strategies are required to prevent or cure relapsed/refractory disease, reduce treatment-related morbidity, improve QoL and outcomes in patients aged ≥60 years. Although curative therapy has been available now for several decades, little information is still known with regard to how HL impacts upon health-related QoL through diagnosis and treatment.

However, it is recognized that the QoL in patients can be significantly affected in patients with HL even prior to the onset of chemotherapy, with substantial differences observed between disease stages (17). It is known that treatment of HL is associated with significant acute and long-term complications (18). In all stages, QoL is observed to be at its worst during chemotherapy with improvement seen quickly thereafter. Many HL patients are in early adulthood, thus maintenance of QoL after treatment completion is crucial. After intensive treatment, QoL can be impaired due to treatment-induced organ dysfunction, psychological problems, fatigue, persisting gonadal and cognitive impairment and social discomfort/difficulties. It has also been demonstrated that QoL was decreased in the long-term (adjusted for age, sex and education status) after curative treatment, significantly and persistently affecting the well-being of survivors (19–22). Economic difficulty and fatigue emerged as being most closely correlated with all affected domains of QoL (23). In particular, persistent fatigue is recognized as one of the greatest challenges faced in patients with HL and consequently the identification of contributing factors and a greater understanding of the patterns of recovery within the various QoL domains is warranted. The detrimental effects of treatment on QoL can severely impact a patient’s return to their normal life. Furthermore, despite significant advances in the treatment of relapsed/refractory HL, patients still continue to have decreased QoL, emphasizing the need to focus on QoL during the initial stages of treatment decision to improve long-term survival (24). Although research shows that QoL ameliorates following treatment, continued negative effects on sexual and psychosocial health as well as chronic fatigue warrant further studies with targeted interventions to mitigate long-term sequela. In addition to seeking improved response rates, awareness and consideration of QoL is recognized as equally important (17).

The assessment of PROs, addressing QoL aspects, symptoms, and treatment satisfaction may yield important additional information to aid in the care of patients. In clinical trials, survival outcomes, clinical response and toxicity are extensively reported while the assessment of treatment on PROs in the sort and long-term is often lacking. The inclusion of humanistic outcomes reported by patients to supplement clinical outcomes is increasingly gaining in importance in HL trials. This ensures that the intended benefits are also based on the patients’ perspective. The measurement of PROs is crucial to understanding the impact of treatment on patients’ physical, psychosocial and functional behavior as well as their symptoms in order to evaluate the risk-reward balance for specific treatments. Thus, the inclusion of PROs as secondary or exploratory endpoints in the design of clinical trials can facilitate cross-comparison across studies that are based on efficacy and patient experience (25). Moreover, the inclusion of PROs can also provide information from the perspective of the patient during and after therapy. The timely addressing of unmet needs that are reported directly by the patient under therapy as well long-term follow-up, can be effective in alleviating the burden of the treatment experience, experiences that may not be easily detected by other means. Currently, the US Food and Drug Administration and European Medicines Agency, recommend PROs as a prioritized treatment outcome (26, 27). Indeed, the added value of including PROs in clinical trials of HL patients has previously been reported (28–30). Data derived from PRO assessment can provide the patient-level impact of regimens on event-free survival, overall survival, and tolerability of the acute as well as the long-term effects of treatment. Furthermore, PROs also yield additional information on benefits/risks of treatments from the perspective of the patient, information that could not have been otherwise obtained from clinician-reported symptomatic adverse events. It is also important to note that improvement in QoL is included in the European Society of Medical Oncology Magnitude of Clinical Benefit Scale to determine the “value” of novel therapeutic options in cancer (31–33). Several systematic reviews on the use of PROs in clinical trials in the HL setting have previously been published (29, 30, 34). However, for trials involving HL patients, it still is unclear as to which PRO instruments are used, frequency of their use, and whether their results are consistently reported. In a recent systematic review, the inclusion of PROs in phase 3 clinical trials in HL including the young adult population between 2007–2020 using the European Organization for Research and Treatment of Cancer Quality of Life Core Questionnaire (QLQ-C30) was reported (34). Analysis revealed that only four trials (17.4%) included PROs, but none of them have yet published the PRO results. Furthermore, there is the lack of data which PRO measures could be recommended as the tools of choice in HL clinical trials. The development of guidelines for the use of PROs in adult patients with hematological malignancies was supported and conceptualized by the European Hematology Association (EHA). The first step is the reporting of this systematic review that explores the use, frequency and consistency of reporting of PRO instruments in randomized controlled trials (RCTs) for HL.

The aim of this systematic review was to evaluate the use and frequency of PROs in RCTs of HL, provide a summary of scales and instruments and evaluate their validity in this patient population.

## 2 Methods

This systematic review followed the 2020 Preferred Reporting Items for Systematic Reviews and Meta-Analyses (PRISMA) updated guideline for reporting systematic reviews (35), and was registered with PROSPERO (CRD42023391552).

### 2.1 Search strategy and criteria for eligibility

An experienced information specialist (IM) developed search strategies to identify RCTs in HL. The database and clinical trial registries searched were MEDLINE (Ovid), Cochrane Central Register of Controlled Trials (CENTRAL), ClinicalTrials.gov, and WHO International Clinical Trials Registry Platform (ICTRP). The searches were conducted for the period from January 1^st^, 2016 until May 31^st^, 2022. The full search strategies are provided in the **Supplementary Material**.

Eligible types of publications were full text-articles, conference abstracts and registry entries of RCTs (including cross-over trials and trials with open-label extensions if initial treatment was continued after study completion). EndNote20^®^ was used for reference management in line with the published guidance (36). We excluded trials performed in mixed hematologic or hemato-oncologic malignancy patient populations. Language restrictions were not applied.

Inclusion criteria were pre-defined as any patient diagnosed with HL regardless of the stage of the disease, age, gender, ethnicity, setting or country. No restrictions were applied based on the investigational or comparator interventions received and included among other comparisons of drug regimens, drug combinations, radiation therapy, radio-chemotherapy, sequential chemo- and radiotherapy, educational or lifestyle interventions as well as alternative medicine interventions. We included RCTs irrespective of the outcomes assessed. However, reports of trials only presenting biochemical or prognostic factors and models were in part excluded.

After data extraction, which aimed primarily at identifying instruments and scales used for the measurement of PROs in HL RCTs, we performed a search for validation studies of the PROMs detected, to their psychometric validity in the HL patient population. In addition, we carried out hand searches for instrument manuals and for any other information not available in the validation studies.

### 2.2 Data collection

**Figure 1:**
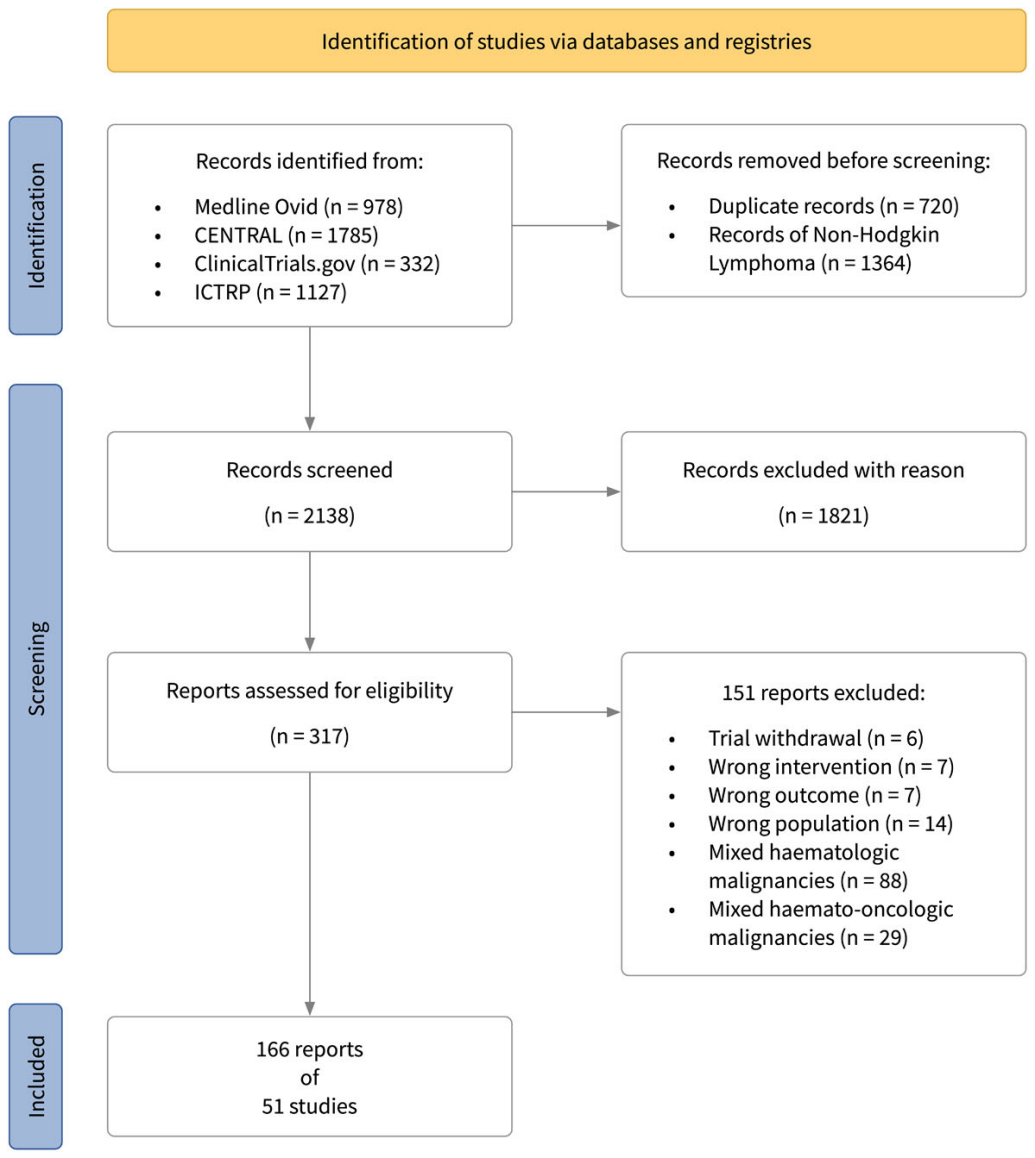
PRISMA flow diagram.

Three researchers (AO, JS, MC) independently screened results of the search strategies for potential inclusions. Any discordance during the selection process were resolved by discussion. The trial and record selection, search counts and reasons for exclusions were recorded in a PRISMA flow diagram (**Figure 1**).

Using a pre-defined template two reviewers (JS, MC) recorded general trial information (trial name, registry number, source, registration date, centricity, completion and publication status), data about the study design (trial phase, blinding, length of follow-up), patient characteristics (patient’s age, disease setting – newly diagnosed or relapsed/refractory, disease stage at diagnosis), investigational and comparator interventions, as well as on the primary and secondary trial outcomes. With the objective to evaluate the prevalence of PROs used as outcome measures (primary, secondary, or exploratory), we assessed whether PROs were planned to be measured, which instruments were planned to be or were actually used, and whether PROs were reported in trial publications. Data extraction followed the guidance of the Cochrane Handbook for Systematic Reviews of Interventions (37). The extraction template was formatted in Excel based on the main outcomes of the review, taking into account the checklists for collecting data published in the Cochrane Handbook for Systematic Reviews of Interventions version 6.3 (38). Missing data was marked as ‘not identified’, but none was rated relevant enough to request unreported data or further details from the study authors.

After the identification of PROMs, we assessed whether global and disease-specific tools were validated in the HL population. Manuals of symptom-oriented tools, e.g., tools focusing on chemo- or radiotherapy induced side effects such as nausea or fatigue, were assessed with regard to the tools’ eligibility for the HL specific disease and treatment setting.

This systematic review aimed to provide a comprehensive summary of PRO instruments across trials and not analyze trial results themselves. Thus, we did not plan or conduct a formal risk of bias assessment. Neither did we record the effect measures reported in trials.

### 2.3 Data synthesis

We conducted a narrative synthesis of findings across trials, summarizing qualitative variables by level frequency (number, %) or in form of graphs and plots. We analyzed trials with regards to their status of completion, publication, and protocol accessibility. The utilization of PROMs was analyzed with respect to the type of instrument (generic, disease-specific, or symptom-specific) and number of PROMs used or planned for use in single trials. We recorded the phase of clinical investigation, and treatment setting (chemotherapy, radiotherapy, stem cell transplantation, supportive treatment measures or a combination) as well as information on the follow-up period. For the assessment of PRO reporting consistency, we compared study registry entries and, where available, protocols with full-text publications and evaluated if PROMs listed in the registry or protocol were reported, partly reported, or not reported in the related publication(s). In case no full text publication was available we proceeded with planned PROMs.

## 3 Results

### 3.1 Search results and included studies

Overall, 4,222 records were identified through database and registry searches. After full-text screening, 166 reports of 51 ongoing or completed trials were included (**Figure 1**). Based on registry entries at the time of data extraction 21 (41%) trials were completed, 25 (49%) were still ongoing. Thereof, 9 trials (18%) were active but not recruiting, 16 (31%) trials were at the recruitment stage, four (8%) studies had an unknown trial status, and one (2%) trial was terminated due to poor recruitment. Publications in full-text/abstract form were available for 33 (65%) trials. Thirty-six studies (70%) were undertaken in the newly diagnosed disease setting, the majority in patients with advanced HL. Fourteen (28%) trials were performed in patients with relapsed or refractory disease. In one trial (2%) the disease setting was not specified. Approximately half (27; 53%) of trials, investigated drug regimens, 19 (37%) trials investigated drug regimens combined with radiotherapy, two trials (4%) exclusively investigated the application of radiotherapy and in three trials (6%) supportive interventions (specifically, one trial investigating a drug mitigating cytotoxic effects of chemotherapy, one trial of antiemetic therapy, and one trial of physical exercise interventions) were studied. In trials with published follow-up data (32 trials, 63%), the median follow-up was 5.2 years. See the **Supplementary Material** (**Supplementary Table 1**) for a summary of included RCTs.

### 3.2 PRO assessment and PROM validation

**Figure 2:**
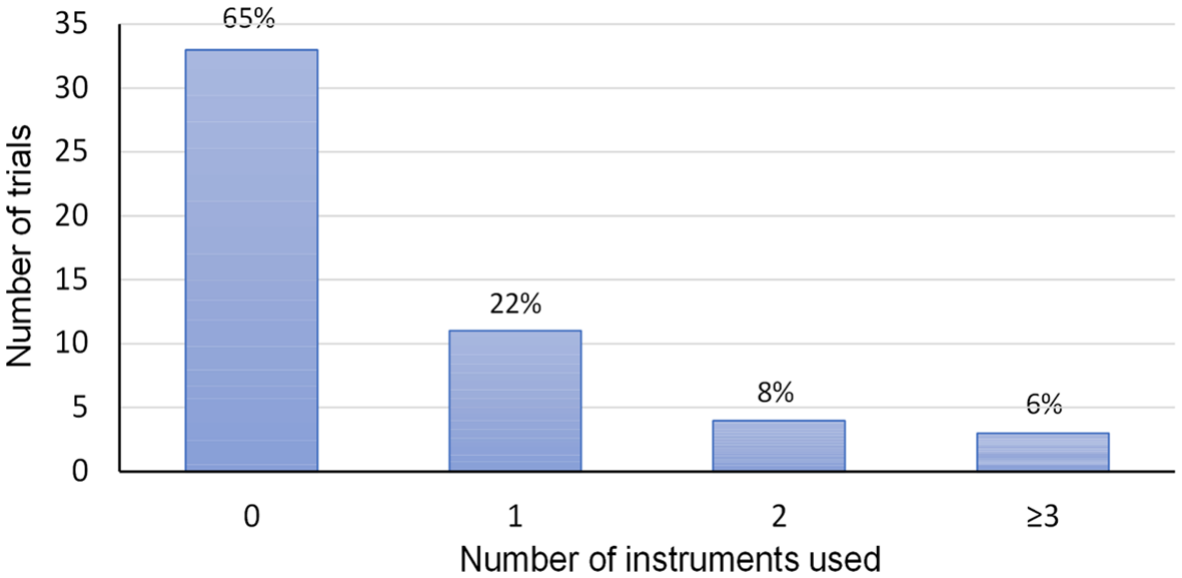
Number of PRO instruments used per trial.

Across trials, ten different PRO measures were reported. Thereof, three were global or disease-specific and seven symptom-specific tools. In 18 trials (35%), completed/ongoing, PRO assessment was mentioned either in their registry entries, protocol, or publications (**Table 1**). Only one trial assessed PRO as a primary outcome, while all other trials evaluated PROs as a secondary/exploratory outcome. Most trials which referenced PRO assessment were phase 3 trials (78%), while fewer trials referencing PROs were phase 1/2 or phase 2 trials (22%). The majority of the 18 trials which considering using PROs, 11 (61%) planned to use one instrument 4 trials (22%) planned to use 2 instruments, and 3 trials more than 2 (three, four, or six instruments respectively) (**Figure 2**).

**Table 1:** PROMs in trials with PRO assessment.

Trial name or Author/Year	Registration number	PRO reported	Global health PROM	Symptom- oriented PROM	Symptom assessed
**Mohammed 2018**	–	+	–	FLIE-tool	Nausea
**-**	CTRI/2020/12/030132	trial ongoing	NS	NS	
**HD14**	ISRCTN04761296	+	EORTC-QLQ-C30	–	
**HD15**	ISRCTN32443041	+	EORTC-QLQ-C30	–	
**HD13**	ISRCTN63474366	+	EORTC-QLQ-C30	–	
**HD6**	NCT00002561	NR	EORTC-QLQ-C30, NS (trial-specific checklist of Hodgkin disease)	–	
**Lysa EORTC 20012**	NCT00049595	NR	EORTC-QLQ-C30	–	
**HD18**	NCT00515554	NR	NS	NS	
**HD16**	NCT00736320	NR	EORTC-QLQ-C30	–	
**HD-R3i**	NCT01453504	NR	NS	NS	
**ECHELON-1**	NCT01712490	+	EORTC-QLQ-C30, EQ-5D	FACT-Ntx, FACIT-Dyspnea	Neurotoxicity, Dyspnea
**AHOD1331**	NCT02166463	NR	CHRIs-Global Scale	FACT-Ntx	Neurotoxicity
**HD21**	NCT02661503	trial ongoing	EORTC QLQ‐C30	EORTC-QLQ-FA13, EORTC-QLQ-CIPN20	Fatigue, neurotoxicity
**Keynote-204**	NCT02684292	+	EORTC-QLQ-C30, EQ-5D	–	
**FIL-Rouge**	NCT03159897	no publication	EORTC-QLQ-C30, EQ-5D	–	
**-**	NCT03712202	trial ongoing	EORTC QLQ‐C30	–	
**SWOG S1826**	NCT03907488	trial ongoing	PRO-CTCAE	–	
**HD11**	NCT05180097	trial ongoing	EORTC-QLQ-C30, EQ-5D, FACT-Lym, PRO-CTCAE	FACT-Ntx, FACIT-Cost	Neutrotoxicity, financial toxicity

NR, not reported; NS, not specified; Ntx, neurotoxicity. "–" means not included and "+" means reported (for column 3, “PRO reported”).

**Figure 3:**
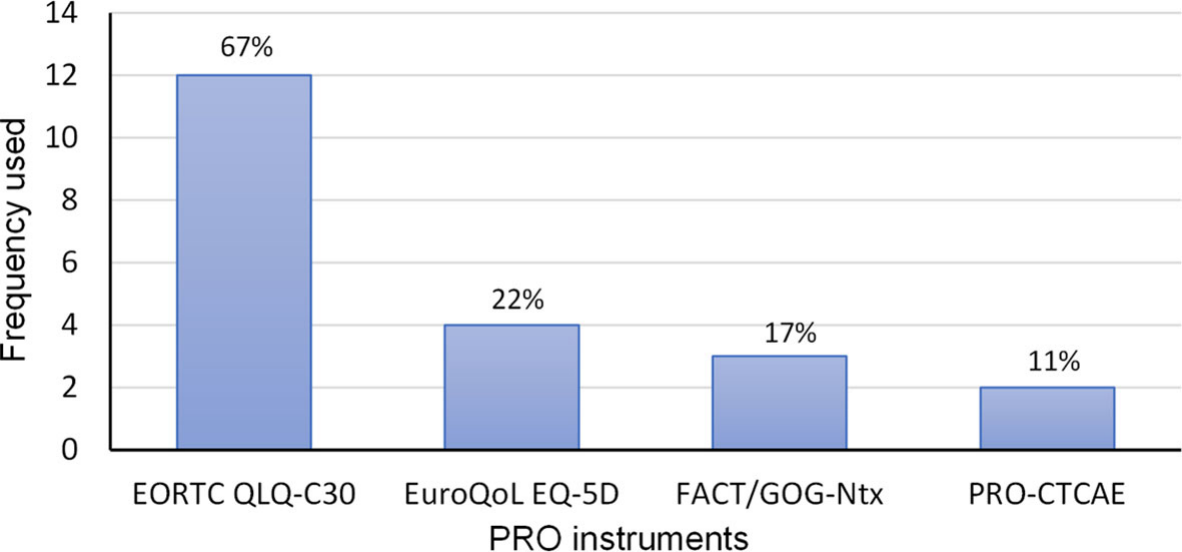
Most frequently used global or disease-specific and symptom-specific tools.

The most frequently used PRO instrument (12 trials, 67%) was the global EORTC QLQ-30 questionnaire (**Table 2**; **Figure 3**), which measures QoL in cancer patients (39), and is validated in a small population of HL patients (40). EuroQol EQ-5D, a questionnaire often used in cost-effectiveness analyses and the calculation of quality-adjusted life years (QALYs), was the second most frequently used PRO instrument (4 trials, 22%). PRO-CTCAE, a global comprehensive tool consisting of 124 items designed for the detection of symptomatic adverse events in cancer patients (41, 42), was applied in two trials (11%). The lymphoma-specific instrument FACT-Lym was planned to be used in one trial. FACT-Lym was developed and originally validated in Non-HL patients (43), and later on validated in a sample of Greek newly diagnosed HL patients (44). One RCT in children and adolescents with HL used the Child Health Ratings Inventory (CHRIs – Global Scale) (45, 46). Of the symptom-specific instruments, FACT/GOG-Ntx was the most utilized tool, being applied in three trials (17%). It allows the assessment of symptoms of chemotherapy-induced peripheral neuropathy (47, 48), and has been validated in lymphoma patients (49). Other symptom-specific tools mentioned in single trials were the EORTC QLQ-CIPN20 (chemotherapy-induced peripheral neuropathy), EORTC QLQ-FA13 (fatigue), FACIT-Dyspnea, FACIT-COST (financial distress of cancer patients), and FLIE (nausea and vomiting). In four trials (22%), which where ongoing or did not report on PROs in their publications, the PROMs applied could not be discerned.

**Table 2:** Overview of PROMs and validation or eligibility status.

Instruments	Frequency of (planned) assessment in HL trials
Global or disease-specific	
EORTC QLQ-C30	12
EuroQoL EQ-5D	4
PRO-CTCAE	2
FACT-Lym	1
CHRIs – Global Scale	1
Symptom-specific	
EORTC QLQ-CIPN20	1
EORTC QLQ-FA13	1
FACIT-Dyspnea	1
FACT/GOG-Ntx	3
FACIT-Cost	1
FLIE	1
Not specified	4

### 3.3 Reporting consistency

In terms of reporting consistency, out of 18 trials with planned PRO assessment, 12 trials (67%) had published trial results. Thereof, six trials (50%), reported on PROs in their publications. In detail, four trials that were completed and reported on PROs did so by using one PROM, as specified in advance (in three trials the EORTC QLQ-C30 and in one trial the FLIE-tool was used). The other two trials with published results reported PROs using two different instruments, namely the EORTC QLQ-C30 and the EQ-5D. In addition, one trial planned to assess PROs using symptom-specific PROMs (FACT/GOG-Ntx and FACIT-Dyspnea). However, one out of the two trials reported PROs incompletely. The remaining six trials with fully published results, did not report on PROs in their publications.

## 4 Discussion

This review shows that only one third of RCTs in HL, completed or ongoing, plan the assessment of PROs. In trials with planned PRO evaluation and available full publications, only half report patient-reported outcomes at least in part. Notably, aside from one HL trial, where PRO was the primary outcome, none of the RCTs that reported on QoL aspects did so in their primary or main trial publication. These results reflect previous findings of underreporting PROs and QoL endpoints in hemato-oncologic trials (50, 51). The most utilized tool for the global assessment of QoL was the EORTC QLQ-C30 questionnaire in two thirds, followed by the EuroQoL EQ-5D in one fifth of Hodgkin RCTs that planned PRO assessment. Aside from one ongoing trial, referencing the FACT-Lym instrument, none of the RCTs planned the assessment of QoL using a disease-specific PROM. The EORTC QLQ-HL27, a validated PROM that was specifically designed for the use in HL trials in conjunction with the EORTC QLQ-C30 was neither mentioned in completed trials as was to be expected, but surprisingly, was neither mentioned in trials registered after the tools’ introduction in 2018 (52). Among the most common symptom-specific tools utilized in HL RCTs were instruments for the assessment of neurotoxicity, like the FACT/GOG-Ntx and the EORTC QLQ-CIPN20, in four out of 18 trials with planned PRO evaluation. In all four trials, the investigational drug regimen contained brentuximab vedotin, an antibody-drug conjugate (ADC) with the known proclivity to cause peripheral neuropathy (53).

The present systematic review clearly identifies the under-reporting of PROMs in clinical trials. Under the auspices of the European Hematology Association, we have recently published guidelines for the use and reporting of PROs in multiple myeloma trials (54) and similarly we are currently developing guidelines for use and reporting of PROs in clinical trials in lymphoproliferative neoplasms. Indeed, in this forthcoming comprehensive guideline, the choice of standardized PROMs and guidance on the use of digital tools for data collection will also be described in detail.

It should be noted that the use of PROs in the framework of clinical trials is distinctly different from the use in clinical practice, in which the use of single items (not domains or scales) are essential to evaluate individual patients experience and needs. Instead, in clinical trials, emphasis is placed on standardized PROMs, scores or scales and the evaluation of statistical or meaningful differences.

A limitation of this review is the introduction of bias due to the underreporting and reporting delays of QoL outcomes. Thus, the results presented can only be seen as an analysis of a point in time, and do not necessarily imply that PROs of a trial would not be reported at a later stage. Furthermore, considering that PROs are oftentimes published in the form of conference abstracts, as part of the **Supplementary Material** or in lesser-known journals it becomes easier to overlook records of PRO-publications, possibly leading to an underestimation of PRO reporting and consistency. Another limitation is that trial protocols were occasionally behind paywalls or not accessible while the trial was ongoing, which might have led to an oversight of planned PRO assessment, if trial entries in registries were incomplete.

In conclusion, a way to overcome the shortcoming of underreporting, QoL and PRO assessment should be made a prioritized trial outcome (i.e. a primary outcome).

Emphasis on QoL in HL patients following treatment may provide important information to facilitate treatment decisions and long-term survival goals.

Future research including prospective, longitudinal randomized trials across both treatment and time are warranted. QoL can be improved by the development of novel, more effective but less toxic therapies and should play an central part of decision-making in HL. The improvement of QoL in patients with HL is an important treatment goal and the inclusion of PROs into routine clinical and research practice has the potential of improving treatment outcomes.

Guidance for the assessment of PROs is necessary to inform on comprehensive outcomes important to patients.

## Author’s note

This systematic review was performed as part of the EHA guideline project on patient-reported outcomes in hematology.

## Data availability statement

The original contributions presented in the study are included in the article/**Supplementary Material**. Further inquiries can be directed to the corresponding author.

## Author contributions

EO: Conceptualization, Supervision, Writing – review & editing. TI: Conceptualization, Formal analysis, Writing – original draft, Writing – review & editing. EL: Conceptualization, Formal analysis, Methodology, Writing – review & editing. MC: Data curation, Formal analysis, Writing – original draft, Writing – review & editing. JS: Data curation, Formal analysis, Writing – original draft, Writing – review & editing. KB: Writing – review & editing. IM: Formal analysis, Writing – review & editing. AO: Data curation, Writing – review & editing. NS: Methodology, Writing – review & editing. SS: Conceptualization, Formal analysis, Methodology, Writing – review & editing.
