# Supplementary material for: Patient-reported outcomes in Hodgkin lymphoma trials: a systematic review
Source: Front Oncol. 2024 Mar 13;14:1353101. doi: 10.3389/fonc.2024.1353101 (PMC10965683; doi:10.3389/fonc.2024.1353101)
Supplement: Supplementary file 1 [file Table_1.docx]

Supplementary Material

# Supplementary Data

## Search strategies for RCTs Investigating Hodgkin Lymphoma

### Cochrane Central Register of Controlled Trials (CENTRAL, 2022, Issue 05)

Search date: May 31^st^, 2022

| # | Search |
| --- | --- |
| 1 | MeSH descriptor: [Lymphoma] this term only |
| 2 | MeSH descriptor: [Hodgkin Disease] explode all trees |
| 3 | germinoblastom*:ti,ab,kw |
| 4 | reticulolymphosarcom*:ti,ab,kw |
| 5 | (hodgkin* or hogkin* or hodkin* or hodgin*):ti,ab,kw |
| 6 | (malignan* near/2 (lymphogranulom* or granulom*)):ti,ab,kw |
| 7 | #1 or #2 or #3 or #4 or #5 or #6 |
| 8 | #7 with Publication Year from 2016 to 2022, in Trials |

### Medline (via Ovid) and Epub ahead of print, in-process, in-data-review and other non-indexed citations, daily and versions

Search date: May 31^st^, 2022

| # | Search |
| --- | --- |
| 1 | *Lymphoma/ |
| 2 | exp Hodgkin Disease/ |
| 3 | germinoblastom*.tw,kf. |
| 4 | reticulolymphosarcom*.tw,kf. |
| 5 | hodgkin*.tw,kf. |
| 6 | ((malignan* adj2 granulom*) or (malignan* adj2 lymphogranulom*)).tw,kf. |
| 7 | (reed sternberg adj2 diseas*).tw,kf. |
| 8 | or/1-7 |
| 9 | randomized controlled trial.pt. |
| 10 | controlled clinical trial.pt. |
| 11 | randomi?ed.ab. |
| 12 | placebo.ab. |
| 13 | clinical trials as topic.sh. |
| 14 | randomly.ab. |
| 15 | trial.ti. |
| 16 | or/9-15 |
| 17 | exp animals/ not humans/ |
| 18 | 16 not 17 |
| 19 | clinical trial, phase iii/ |
| 20 | ("Phase 3" or "phase3" or "phase III" or P3 or "PIII").ti,ab,kw. |
| 21 | (19 or 20) not 17 |
| 22 | 18 or 21 |
| 23 | 8 and 22 |
| 24 | limit 23 to dt=20160101-20220531 |
| 25 | remove duplicates from 24 |

### ClinicalTrials.gov

Search date: May 31^st^, 2022

| Search |
| --- |
| hodgkin* \| Interventional Studies (2016-) |

### WHO International Clinical Trials Registry Platform (ICTRP)

### Search date: May 31^st^, 2022

| Search |
| --- |
| hodgkin* (2016-) |

# Supplementary Tables

## Supplementary Table 1. Summary of included RCTs

| Trial name  or Author/Year | Registration  number | Trial status  (trial registries) | Publication available | Treatment setting | Primary outcome | PRO  planned | PROs reported |
| --- | --- | --- | --- | --- | --- | --- | --- |
| P9426 | - | completed | + | ND | EFS | - | - |
| Mohammed 2018 | - | completed | + | ND | QoL | + | + |
| - | CTRI/2020/08/027495 | recruiting | - | ND | EFS | - |  |
| - | CTRI/2020/12/030132 | recruiting | - | ND | FFP | + | trial ongoing |
| HD14 | ISRCTN04761296 | completed | + | ND | FFTF | + | + |
| HD15 | ISRCTN32443041 | completed | + | ND | FFTF | + | + |
| HD13 | ISRCTN63474366 | completed | + | ND | FFTF | + | + |
| HD6 | NCT00002561 | completed | + | ND | Survival | + | NR |
| EORTC-GELA H9 | NCT00005584 | unknown | + | ND | CR/CRu rate | - | - |
| AHOD0031 | NCT00025259 | completed | + | ND | EFS | + | NR |
| Lysa EORTC 20012 | NCT00049595 | completed | + | ND | EFS | + | NR |
| HD12 | NCT00265031 | completed | + | ND | FFTF | - | - |
| EORTC/LYSA/IIL H10 | NCT00433433 | unknown | + | ND | PFS | - | - |
| EuroNet‐PHL‐C1 | NCT00433459 | completed | + | ND | EFS | - | - |
| HD2000 | NCT00443677 | completed | + | ND | PFS, secondary malignancy,  late toxicity | - | - |
| HD18 | NCT00515554 | completed | + | ND | PFS | + | NR |
| - | NCT00654732 | completed | abstract only | ND | EFS | - | - |
| RATHL | NCT00678327 | active, not recruiting | + | ND | PFS (at 3 years) | - | - |
| HD16 | NCT00736320 | unknown | + | ND | PFS | + | NR |
| FIL HD0801 | NCT00784537 | completed | + | ND | EFS | - | - |
| GITIL/FIL HD0607 | NCT00795613 | unknown | + | ND | PFS | - | - |
| RAPID | NCT00943423 | active, not recruiting | + | ND | PFS | - | - |
| AETHERA | NCT01100502 | completed | + | R/R | PFS | - | - |
| - | NCT01169636 | completed | + | R/R | CR, MTD | - | - |
| IIL | NCT01251107 | completed | + | ND | FFP | - | - |
| HD17 | NCT01356680 | completed | + | ND | PFS | - | - |
| AHL2011 | NCT01358747 | completed | + | ND | PFS | - | - |
| HD-R3i | NCT01453504 | terminated | + | R/R | CRR | + | NR |
| - | NCT01569204 | completed | + | ND | CRR | - | - |
| ECHELON-1 | NCT01712490 | active, not recruiting | + | ND | mPFS | + | + |
| - | NCT01896999 | recruiting | - | R/R | CRR | NI |  |
| AHOD1331 | NCT02166463 | active, not recruiting | + | ND | EFS | - | - |
| HD21 | NCT02661503 | recruiting | - | ND | PFS, TRM | + | trial ongoing |
| Keynote-204 | NCT02684292 | active, not recruiting | + | R/R | PFS, OS | + | + |
| EuroNet-PHL-C2 | NCT02684708 | active, not recruiting | - | ND | EFS | NI |  |
| NIVAHL | NCT03004833 | active, not recruiting | + | ND | CRR | - | - |
| FIL-Rouge | NCT03159897 | active, not recruiting | - | ND | PFS | + | no publication |
| - | NCT03250962 | recruiting | + | R/R | TRAEs, CRR, DOcR | NI |  |
| - | NCT03712202 | recruiting | - | ND | PFS | + | trial ongoing |
| SWOG S1826 | NCT03907488 | active, not recruiting | - | ND | PFS | + | trial ongoing |
| - | NCT04044222 | recruiting | - | R/R | PFS | NI |  |
| - | NCT04342936 | recruiting | - | R/R | PFS | NI |  |
| BRESELIBET | NCT04378647 | recruiting | - | R/R | PET-CT results | NI |  |
| - | NCT04486391 | recruiting | - | R/R | PFS | NI |  |
| - | NCT04514081 | recruiting | - | R/R | ORR, TRAEs | NI |  |
| - | NCT04636255 | recruiting | - | NI | Peak oxygen consumption | NI |  |
| RADAR | NCT04685616 | recruiting | - | ND | PFS | NI |  |
| HD11 | NCT05180097 | recruiting | - | R/R | CR | + | trial ongoing |
| - | NCT05244642 | recruiting | - | R/R | PFS | - |  |
| FIL - A-BEGEV | NCT05300282 | not yet recruiting | - | R/R | CRR before ASCT | NI |  |
| LYSA H34 | RECF0219 | completed | + | ND | EFS | - | - |
| *Abbreviations:* ND – newly diagnosed, R/R – relapsed and/or refractory, NI – no information, NR – not reported, QoL – quality of life, EFS – Event-free Survival, PFS – Progression free survival, FFP – freedom from progression, FFTF – freedom from treatment failure, OS – overall survival, CR – complete response, CRR – complete response rate, ORR – overall response rate, DOcR – duration of complete response, TRAE – treatment related adverse events, TRM – treatment related mortality | | | | | | | |
